# Leeches

**Published:** 1881-06

**Authors:** 


### SELECTIONS.

### LEECHES.

Leeches have been used from time immemorial as the best means of local depletion. Their uses are described by all the Latin and Arabian medical writers, as well as the mode of applying and preserving them. In Greek medicine they were not known apparently until a date, the first mention of them being made by Themison, who lived in the century preceding the Christian era.

Leeches are indeed a powerful therapeutic agent, and give to the practitioner in many instances a control over disease which he could not otherwise obtain. The great use made of them in modern practice has occasioned them to become a considerable article of commerce, and attempts have been lately begun in Europe to propagate them on a large scale, this being done by means of natural meadows, in which numerous small ponds are constructed, where the leeches, with certain precautions as to nourishment and preservation, multiply and grow so rapidly as to become a source of profit. To be profitable these leeches must multiply by the million; to do this they must be liberally supplied with food. For this purpose speculators buy up all the old and worn-out horses, and drive or drag them into the ponds, which are subdivided by wooden compartments, so placed that when the poor animals have been forced into the mud there is no escape for them. The leeches fasten on the horse by thousands. In a few moments he is black with the hungry bloodsuckers, which fix themselves most of all on the open wounds and galls incurred in his many years of service. The frantic terror of the poor beast is terrible. Bleeding from all the most sensitive parts, he tries vainly to shake off the leeches, but at lasts sinks down into the slime and is seen no more. But though the sufferings of the horses are not sufficient to check the barbarities of greedy speculators, yet an argument should be heard against leeches so nourished, which are very likely to covey poisonous and infecting products to the human system.

The proper preservation of leeches is an object of importance to the physician, as they are liable to a great and sudden mortality. If they are kept in moist earth, turf or clay, they should be examined from time to time and dead ones removed. The water should be changed at least once a week, and oftener in warm weather. Thus it will be observed that the conditions for the preservation of leeches are pure air, pure water and a uniform temperature. The usual food of the leech consists of animal or vegetable infusoria, which it swallows in large quantities, as the whale swallows a mass of squid. But while the whale ejects the swallowed water by the nostrils, and only retains the squid in his stomach, the leech gives off the superfluous water by a sort of perspiration through a system of glands in the outer skin and retains the infusori in the stomach. It has a great love for the blood of both cold and warm-blooded animals, preferring however the latter, and often gorging itself so much that it cannot digest it, but dies. Not every variety of leech is adapted for medicinal use. While their employment as a means of abstracting blood was more or less practiced in ancient times, it is only within a comparatively recent date that they have been used in large quantities. Three varieties are used; the Swedish or German leech is considered the best the Hungarian next and the American the poorest variety.

Large leeches should be used only when blood is to be freely abstrated and when the part is not very sensitive or exposed. The wounds they make are liable to leave visible scars. Small leeches are always preferable for application to the face or neck, for the reasons just given and also because the bleeding from the wounds is more easily controlled.

